# Supplementary material for: Are carbon emissions trading and green financial instruments synergistic? -Comprehensive quantitative research based on content analysis
Source: PLoS One. 2024 Mar 7;19(3):e0298601. doi: 10.1371/journal.pone.0298601 (PMC10919601; doi:10.1371/journal.pone.0298601)
Supplement: S1 Data — (ZIP) [file pone.0298601.s001.zip › Data/地级市Z_F工作报告.zip!/╔╜╬≈/╦╖╓▌╩╨/└┤╫╘╣1⁄2╓┌║┼í╛┬φ┐╦╩2╛▌═°í┐_2004.docx]

各位代表：

　　本届政府任期已满。现在，我代表市人民政府向大会作报告，请予审议，并请市政协委员和其他列席人员提出意见。

　　一、关于过去五年的政府工作

　　五年来，我们在中共朔州市委的领导下，高举邓小平理论伟大旗帜，全面贯彻“三个代表”重要思想，团结和依靠全市人民，开拓创新，争先发展，圆满实现了市三届人大一次会议提出的经济社会发展目标，全市呈现出改革深化、开放扩大、经济发展、社会稳定的良好局面。

　　经济发展跃上新台阶。扭转了上世纪末煤炭工业严重滑坡、1999年地区生产总值和财政收入负增长的被动局面，战胜了去年突如其来的“非典”疫情，全市经济从建市以来的最低谷逐步走上健康快速发展的轨道，成为历史上最好的发展时期之一。综合经济实力明显增强。地区生产总值由1998年的79.8亿元增加到2003年的118．6亿元，年递增6．1％，近两年保持了13％以上的高增长。财政总收入由1998年的6.41亿元增加到2003年的14．71亿元，净增8．3亿元，年递增10.5％，近两年分别增长23．15％和18.44％，固定资产投资累计145亿元，比建市头10年总投资额多63．52亿元，比上一个五年增长2．5倍。农业和农村经济全面发展，雁门关生态畜牧经济区建设起步良好。在大面积退耕还林还草的情况下，粮食产量稳定在60万吨左右；畜牧业发展势头强劲，猪、牛、羊饲养量年均增长3．6％、9．2％和9％，其中奶牛达到10万头，净增3．7万头。工业经济速度效益同步提升。规模以上工业增加值达到42．1亿元，年均增长13．63％；实现利税13.36亿元，为1998年的16．5倍，年递增75.21％。商贸经济繁荣稳定。社会消费品零售总额年均增长10.8％。人民生活水平不断提高。城镇居民人均可支配收入达到7057元，净增2640元，年均增长9．8％；农民人均纯收入达到2521元，85．8％的农村人口基本达小康。

　　改革开放呈现新局面。农村完成了土地二轮延包，税费改革基本结束。企业改革坚持抓重点、破难点，全面推进。地方国有企业改制面达到95％，地方煤矿改制面达到87％，一大批困难企业走出困境。市、县两级机构改革和撤并乡镇工作顺利完成。社会保障体系建设扎实推进。“两个确保”和城市居民最低生活保障实现应保尽保，并彻底清理了历史拖欠的离退休职工养老金。对外开放进一步扩大，招商引资成效明显。引进国内外大企业、大集团20多家，签订合同投资73亿元，已完成28亿元。海关进出口总额由1998年的618万美元增加到1752万美元，年递增23.2％。

　　结构调整实现新突破。共铺开调产项目188个，总投资2O8亿元。到2003年底已建成投产94个，年新增销售收入25亿元，新增利税4.5亿元，增加就业岗位3万多个。全市三大产业比例由1998年的18．5：49．3：32．2演进为2003年的13．1：50．9：36。农业特色产业正在形成。种植结构全面优化，园区养殖、规模养殖蓬勃兴起，一批龙头企业成长壮大，农业产业化经营初具规模。工业多元支柱产业格局正在形成。五年新增煤炭生产能力2000万吨，新增煤炭筛选能力2000万吨，新增发电量40亿千瓦时，新增乳品生产能力20万吨，新增日用瓷生产能力4亿件，冶金、化工、食品等行业的生产能力也有大幅度提高。旅游业和新兴服务业正在成为经济增长的新亮点。多成份的所有制结构正在形成。非国有经济比重由1998年的15．3％提高到52．8％，民营经济占到地方经济总量的70％以上，一批民营企业在结构调整中显示出主力军作用。

　　基础设施建设取得新成效。生态建设力度加大。实施了京津风沙源治理、首都水资源可持续利用、退耕还林等一批国家和省级重点工程，累计完成人工造林、封山育林120多万亩，其中退耕还林83．7万亩。基本农田建设新增节水面积20万亩，水土保持治理55万亩。实施人畜饮水解困工程，解决了25万人、6万头大牲畜的严重缺水问题。公路建设跨越式推进。建成了大运高速公路朔州段、世纪大道等一批重点工程。公路通车里程达到4805公里，每万人拥有公路里程居全省第一。村通油路去年一年建成1373公里，超过建国50多年的总和，全市78％的行政村通了油路。城市建设与管理水平进一步提高。完成了《城市总体规划》修编草案。加快了路、水、电、气、热、绿地等城市基础设施建设和生态环境建设，完成了二水厂一、二期工程、崇福寺文脉工程、城北生态工程、七里河公园续建、体育广场、北关路改造、市二中、市三小等二十多项重点工程，城市人均公共绿地、人均道路、人均住房面积名列全省前茅，先后两次被建设部评为城市环境综合整治先进市和全国园林绿化先进城市。电力、邮电、通讯事业发展迅猛。城市功能日趋完善，城市品位大为提高。软环境整治全面展开，投资环境进一步改善。

　　社会事业得到新发展。科技工作面向经济建设，组织实施市级以上科技项目295项，取得各类科技成果132项，科技进步对经济增长的贡献率由1998年的38.8％提高到42％。教育事业迅速发展。“两基”水平不断巩固提高，“普九”人口覆盖率由1998年的88％增加到97％，农村劳动力非文盲率达到98％以上。职业教育、成人教育发展较快。教育质量稳步提高，教育工作综合考评和高考达线率一直位居全省前列。城乡卫生工作得到加强。基层医疗卫生设施有所改善，公共卫生体系建设有序进行，地方病、传染病得到有效控制。广播电视圆满实现“村村通”。计生工作切实加强，人口计划圆满完成。环境保护力度加大，环境恶化趋势得到有效遏制。民政、宗教、人防、地震、气象、档案、老龄、残疾人等工作都取得新成绩。

　　精神文明和民主法制建设迈出新步伐。思想道德建设继续加强，学习宣传邓小平理论和“三个代表”重要思想活动深入开展。精神文明创建活动卓有成效，连续五年被评为全省文明城市创建先进市。文化体育活动空前活跃，人民群众精神生活更加丰富多彩。民主法制建设得到加强。市政府认真执行市人大及其常委会的决定和决议，自觉接受人大监督，并积极听取市政协、各民主党派、人民团体及各界群众的意见，五年办理人大代表意见、建议和政协委员提案近千件。政府职能进一步转变，行政审批制度改革有序推进，依法行政水平明显提高。圆满完成了“三五”普法教育，“四五”普法全面启动。严打整治逐步深入，社会治安综合治理成效明显。廉政建设不断加强，消极腐败现象和行业不正之风得到有效惩治。政务公开的经验在全国、全省推广。

　　各位代表，过去五年，是全市上下进一步解放思想、锐意进取的五年，是经受住各种困难考验、阔步前进的五年，是各项事业蒸蒸日上、人民生活显著改善的五年。朔州能有今天的局面，确实来之不易。这是市委坚强领导，市人大有效监督，市政协、各民主党派和人民团体鼎力支持，全市人民艰苦拼搏、不懈努力的结果。在此，我谨代表市人民政府，向为朔州改革开放和现代化建设作出重大贡献的全市人民，向所有关心支持朔州建设的驻朔单位、人民解放军、武警官兵及各界的同志们、朋友们，表示崇高的敬意和衷心的感谢！

　　五年的实践，使我们深刻地体会到，只有全面落实“三个代表”重要思想，坚持立党为公，执政为民，真心实意为群众办实事，才能真正维护和实现广大人民群众的根本利益；只有把发展作为第一要务，坚持结构调整这一主线，才能真正把朔州的经济建设推上持续快速健康发展的轨道；只有大力推进体制创新，坚持建立和完善市场经济体制，才能真正为经济和社会发展注入不竭动力；只有切实转变政府职能，坚持优化环境，大力推进对外开放，才能真正提高投资吸引力和经济竞争力；只有实施可持续发展战略，坚持科学的发展观，把控制人口、节约资源、保护环境摆在十分重要的位置，才能真正实现经济和社会的协调发展；只有切实推进精神文明和民主法制建设，努力提高全市人民文明素质和城市文明程度，才能真正为经济和社会发展提供强有力的保障。

　　在肯定成绩的同时，我们也清醒地看到，在经济和社会生活中还存在许多不容忽视的问题。农民收入渠道不宽，农民收入增长缓慢；接替产业的成长步伐还不够快，结构性矛盾仍很突出；深化国企改革和完善社会保障体系的任务仍很艰巨；投资环境还不够宽松；环境污染问题仍很严重；社会治安状况群众不够满意；干部队伍作风建设仍需加强，等等，对此，我们一定要居安思危，高度重视，下决心予以克服和解决。

　　二、关于下届政府工作的建议

　　各位代表，未来五年，是我们全面建设小康社会、加速推进社会主义现代化的关键时期，也是加快建立比较完善的社会主义市场经济体制、全面提升改革开放水平的攻坚阶段。新一届政府的工作任重而道远，必须以邓小平理论和“三个代表”重要思想为指导，认真贯彻党的十六大和十六届三中全会精神，牢固树立全面、协调、可持续的发展观和以人为本、执政为民的理念，坚持农业产业化、工业多元化、城市园林化、政府高效化、人民生活小康化的方向，紧紧围绕“实现新跨越、建设新朔州、经济翻一番、社会大发展”的总目标，切实抓好雁门关生态畜牧经济区和大运高速公路经济带两大战略工程，着力构建比较合理的产业构成体系、发达的科技进步体系、有力的人才支撑体系、完善的市场经济体系、健全的社会保障体系，进一步推进民主政治建设，全力维护社会稳定，推动全市经济社会健康快速发展。

　　落实这一指导思想，非常重要的一点，就是要牢固树立全面、协调、可持续的科学发展观。这既是经济工作必须长期坚持的重要指导思想，也是解决当前经济社会发展诸多矛盾的基本准则。目前，我们已经进入了一个新的增长期，面临许多难得的机遇。国际国内经济形势持续向好，能源原材料需求更趋旺盛，使我市经济增长有了充分的市场支撑；全球性结构调整和产业转移步伐加快，为我市的结构调整拓展了新的空间；国家进一步加强电源电网建设、促进大型煤炭基地建设、着力缓解煤电油运的宏观调控政策，为我市充分发挥能源优势提供了新的契机；经济体制改革的不断深化与完善，也必将带动我市经济发展的活力进一步增强。我们完全有条件、也有能力在今后一个时期内，实现全市经济新的更大的跨越。这是新一届政府的核心任务和义不容辞的职责。但这一新的增长期，要求我们的发展必须是全面、协调、可持续的发展，是经济与社会共同进步，人与自然和谐协调，城乡之间、区域之间统筹兼顾的发展。这是全面建设小康社会的重要内容，也是人类文明进步的必然要求。我们必须按照科学发展观的要求和中央宏观调控的有关政策，坚持物质文明，精神文明和政治文明三个文明建设一起抓，在推动经济快速发展基础上，努力促进社会的全面进步和人的全面发展；坚持在开发利用自然中，努力促进人与自然的和谐相处，实现可持续发展；坚持在确保经济增长速度的同时，更加重视增长的质量和效益；坚持在促进发展的进程中，不仅要关注经济指标，而且要关注社会发展指标、人文指标和环境指标；不仅要增加经济发展的投入，而且要增加促进社会发展、改善环境质量的投入，以进一步拓展发展空间，提高发展质量，实现更好发展。总之，只有坚持科学的发展观，才能确保我们的工作经得起实践的检验，经得起群众的检验，经得起历史的检验！

　　按照科学发展观的要求，今后五年，全市“实现新跨越、建设新朔州、经济翻一番、社会大发展”的目标，应该而且也必须包括这样六个方面的具体内容：

　　一是经济增长更加强劲。各主要经济指标增幅要全面超过建市以来的平均水平，使全市经济增长达到一个新的历史高峰，2008年，地区生产总值达到2400亿元，比2003年翻一番，年递增13％，财政总收入达到31亿元，比2003年增长1．1倍，平均年递增15．8％。固定资产投资累计达到400亿元以上，接近过去五年的3倍，平均年递增17．1％。

　　二是产业结构更加合理。农业和农村经济要实现半农半牧目标，建成全省一流的生态畜牧强市，其中奶牛要突破20万头，鲜奶产量突破70万吨。工业要建成全省最大、全国领先的煤电传统工业基地。到2008年，全市煤炭产量要达到8000万吨，比2003年增加2700万吨，产值突破100亿元。电力装机容量达到500万千瓦，比2003年增加一倍多；发电量达到425亿千瓦时，产值突破100亿元。食品、陶瓷、冶金、化工、材料等新兴产业也要在全省独树一帜，凸显优势。到2008年，全市乳制品产量要达到57万吨，完成产值44亿元左右，年递增55．3％。日用瓷产量达到8亿件，产值达到7亿元，年递增28％左右。冶金行业产值达到38亿元左右，年递增125％；化工业产值达到25亿元，年递增36％左右。同时大力发展旅游、服务等第三产业，形成多元并举的产业格局。

　　三是市场经济体制更加完善。全面完成国有企业改制任务，绝大部分企业建立起比较规范的现代企业制度；国有资产管理体制建立健全。新的投融资体制基本建立，资本、技术、人才等要素市场充分发育，产权交易、物资交流等有形市场不断扩大，市场配置资源的基础性作用进一步发挥。经济外向度明显提高，招商引资取得重大突破，引进资金达到200亿元左右，引进项目突破400项，引进高新技术不少于50项。

　　四是发展环境更加优越。基础设施建设取得更大进展，城市功能进一步完善，城市化水平达到42％左右。生态环境明显改善，大气污染、水污染得到有效控制；全市林木覆被率达到23％，城市绿化覆盖率达到43％，建成国家生态园林城市。软环境得到更大力度的整治，干事创业的外围环境更加宽松顺畅。

　　五是社会文明程度更加提高。科技、教育更快发展。九年义务教育高标准普及，高中教育及其他教育快速推进，科技在经济增长中的贡献率达到47％左右，人才支撑体系建设见到成效。社会事业繁荣兴旺，高标准建成一批卫生、文化、体育设施。精神文明建设扎实推进，进入全国文明城市行列。政府行为规范高效，依法行政水平大为提高。民主法制建设进一步加强，社会治安状况明显改善。

　　六是人民生活更加富裕。城乡居民收入不断增长。2008年，城镇居民人均可支配收入达到1．1万元，比2003年净增3950多元，年均增长9．3％，保持全省领先地位；农民人均纯收入达到3800元，比2003年净增1280元，年均增长8．3％，进入全省前茅。农村基本消灭贫困，农民主体上步入宽裕型小康。就业服务体系进一步健全，城镇下岗职工和新增劳动力的就业水平明显提高。城镇社会保障体系不断完善，达到应保尽保；农村社会保障体系建设取得重大突破，农村最低生活保障制度、义务教育保学金制度、新型合作医疗制度和医疗救助制度要基本建立，切实解决好农民看病难、上学难等问题。

　　这些目标，既是鼓舞人心的，也是极其艰巨的，需要全市人民的团结拼搏、不懈努力，尤其需要各级政府部门的同志身先士卒，抢抓机遇，求真务实，扎实苦干。

　　完成五年目标，今年首先要起好步。今年经济社会发展的主要预期目标为：地区生产总值137亿元，增长13．3％；全部工业增加值61．8亿元，增长19．6％；粮食总产保持在60万吨左右，与去年持平；财政总收入17．36亿元，增长18％；一般预算收入5．9亿元，增长11．7％；城镇居民人均可支配收入7700元，增长9．1％；农民人均纯收入2700元，增长7．1％；社会消费品零售总额44亿元，增长12％；海关进出口总额1930万美元，增长8％；固定资产投资62亿元，增长22．7％；人口自然增长率控制在8‰以内；城镇登记失业率控制在4.5％以内。

　　今年的主要工作包括八个方面：

　　（一）把解决“三农”问题作为重中之重，大力发展农业和农村经济

　　始终把解决“三农”问题摆在工作首位，更多地关注农村、关心农业、关爱农民。进一步强化对农业的支持和保护，继续推进雁门关生态畜牧经济区建设，促进农业和农村经济结构战略性调整，拓宽农民增收渠道。

　　贯彻中央一号文件精神，加大支农护农力度。认真执行国家农业税减免政策，今年减免1个百分点的政策必须落实到位。农民种植小麦、玉米等享受到的840万元省市直补资金，要及时足额兑现到户。合理调整土地出让金使用结构，市县两级土地出让金纯收益的60％必须用于农业发展，其中30％用于土地开发、中低产田改造和高标准农田建设；新增建设用地有偿使用费留市、县部分和土地开垦费必须全部用于耕地复垦开发和地力建设。加大财政支农力度，今年将从市财政预算内资金中拿出1400万元；从预算外资金中拿出1200万元，用于支持农业和农村经济发展。严格控制农业生产资料价格。河灌区水价要基本稳定五年不变。实行粮食最低收购价政策，按照国家和省制定的主要粮食品种最低收购价，制定我市玉米最低收购价，确保粮农收益。实行最严格的耕地保护制度，确保五年内全市耕地面积稳定在400万亩以上，粮田面积稳定在290万亩以上。在此前提下，加大科技兴农力度，挖掘土地潜力、提高单位面积产量，实现粮食增产，并增加市县两级粮食储备，确保粮食安全。

　　加强服务体系建设，推动畜牧业更快发展。我市畜牧业服务体系的极度滞后与脆弱，严重影响和制约了畜牧业的健康发展。市政府决定，今年要积极推进畜牧科技、防疫检疫、畜禽良种繁育、饲草饲料种植加工、畜牧市场信息、畜牧法制保障、畜牧合作八大服务体系的建设，重点实施农校与山西农大联合办学、红旗牧场科技示范园区、建设市级动物疫病监测中心、完善30个乡镇兽医中心站及市奶牛医院等20项工程。这八大体系20项工程，总计投资近1．4亿元，其中需政府投资就达900多万元，不管多大困难，我们都要坚决办成办好。推进规模养殖。重点抓好朔城区金沙生态畜牧园区、平鲁移民新村畜牧园区、山阴城东万亩畜牧园区、怀仁云东畜牧园区、应县大临河畜牧园区及右玉三源等一批畜牧园区。通过体系建设和园区带动，实现畜牧业新的突破，力争今年全市奶牛饲养量达到13万头，比去年增加3万头；肉牛饲养量达到12万头，增长16％；羊发展到230万只，增长11％；农民人均畜牧业收入达到1100元以上，占到农民人均纯收入的40％左右。

　　培育壮大龙头企业，增强辐射带动能力。把进一步做强做大农产品加工业作为全市经济发展的突出亮点来抓，牢固树立“扶持产业化就是扶持农业、扶持龙头企业就是扶持农民”的观念，坚持走产业化经营的路子，重点扶持古城、康喜、雁门、右玉御羊、嘉利淀粉、旺发饲草加工等一批具有较大发展潜力的企业，提高龙头企业对农业增效、农民增收的带动力。

　　继续推进生态建设，努力创建“绿色朔州”。确保退耕还林政策落实到位。继续实施首都水资源可持续利用、京津风沙源治理、退耕还林和通道绿化等重点工程，由点向面扩展。继续抓好大运高速公路绿化、六县区城郊生态森林公园等示范工程建设。抓好以增水节水为主的水利工程和水保治理。在七大河灌区和四大井灌区建设100万亩高效节水农田，新增水保治理面积60万亩。

　　抓好小康建设和扶贫开发，加快共同富裕进程。继续推进农村小康建设，争取年内3个乡镇、57个村、1万人基本达小康，有9个乡镇、130个村、11．7万人步入宽裕型小康行列。大力实施旱作节水、种草养畜、农产品加工和移民搬迁四大扶贫工程，增加贫困农民收入，争取年内解决6个乡、123个村、 3．2万人的温饱问题。

　　（二）继续推进经济结构调整，着力培育更多经济增长点

　　朔州经济发展的潜力在调整，出路在调整。要坚定不移推进结构调整，加快实施“2261”工程，即突出雁门关生态畜牧经济区和大运高速公路经济带建设两大战略重点，做精做优煤电两大传统产业，着力培育食品、陶瓷、冶金、材料、化工和旅游六个接替产业，抓好100多个重点调产项目，确保结构调整明显见效。

　　继续推进行业结构调整。紧紧围绕“传统产业新型化、接替产业规模化”的方向，加快调整步伐。煤炭工业要努力实现开采规模化、生产洁净化、企业集团化。依托大企业的力量，推进一批大型矿井和洗煤、配煤项目建设，力争今年煤炭产量达到6500万吨，入洗能力达到5000万吨。电力工业要抓住电力市场供不应求的有利时机，与大企业通力协作，积极发展大型坑口电厂、矸石电厂和高载能产品自备电厂，力争今年电力装机容量增加到350万千瓦。食品业重点开发乳制品、精细肉、小杂粮、淀粉、饮品和蔬菜六大系列产品，逐步形成规模优势，争取今年乳品产量达到20万吨。陶瓷业重点发展中高档日用瓷，开发建筑瓷、工业瓷，力争今年日用瓷产量达到5亿件。冶金业集中搞好铝硅合金、硅锰合金两大系列产品的开发，推进煤电铝联产联营。材料业要大力开发高岭土、新型墙体材料，积极发展高标号水泥和特种水泥。化工业要重点发展矿药、甲醇、等有机化工，限制发展污染严重的化工产品。旅游业要重点开发大运高速公路黄金旅游带及广武生态文化旅游区、崇福寺文脉工程、应县木塔、右玉杀虎口、怀仁清凉山和平朔露矿六个旅游景点。

　　加快项目建设和园区建设。年初全市共规划101个调产项目，要重中选重，分类指导、择优扶持，加快实施步伐。特别是对一些大项目，如平朔两个500万吨矿井扩建、同煤右玉两座煤矿420万吨矿井扩建和怀仁2X20万千瓦机组、中煤刘家口木瓜界两个500万吨洗煤厂、大唐2X50万千瓦机组，国电怀仁4X60万千瓦机组、晋能铝硅合金、古城及蒙牛雁门乳业液态奶生产线等项目，要紧盯不放，全程服务，快速推进。紧紧依靠体制和机制创新，推动和促进项目建设。坚持政府创环境、企业搞项目的原则，不断完善政府支持方式。在立项审批上给予便利，在项目实施中全方位帮助。创新资金筹措机制。在积极争取上级资金的同时，设立市级调产基金。市政府已筹集3000万元，以后每年都要筹集一定数量资金，作为市级调产基金，采取暂付前期费用、贴息入股和先入后退、滚动发展等方式，支持重点调产项目，以此吸引银行和社会资金。完善市场运作机制。坚持外引内功，鼓励更多的民营企业、外来企业参与项目建设。要把项目建设与加快区域结构调整结合起来，推动更多的调产项目向大运高速公路、平朔一级公路沿线和县城附近的“八大工业园区”（市经济技术开发区、朔城区富甲工业园区、平鲁工业园区、山阴城东工业园区、怀仁云东工业园区和嘉明工业园区、应县城南工业园区、右玉威远工业园区）集聚。正确处理耕地保护与园区建设关系，推动园区建设健康发展。在确保耕地占补平衡的前提下，政府要努力盘活存量土地，提高土地综合利用水平，依法统一征用和储备工业园区发展用地。

　　放手放胆发展非公有制经济。进一步解放思想，加大支持力度，推动非公有制经济大发展、大提高。在政策上，消除一切歧视和体制性障碍。全面放宽市场准入和投资领域，按照“不禁止、则自由”的原则，允许非公有制资本进入法律法规未禁止一切行业和领域，并享受与其他企业同等待遇。放宽注册登记限制，允许新设立的非公有制企业注册资本分步到位，限期补足。在工作上，建立有效的帮扶体系。加强信息传递，搞好项目推荐和引导，扶持发展民营高科技资源深加工和农产品加工等项目。加快信用担保体系建设，为非有制企业的起步发展提供有效支持。有重点地推动成立一些行业协会，加强对非公有制企业的协调和管理。支持、鼓励非公有制企业参与国有经济的改组改制和结构调整，培育一批带动性强的骨干企业。疏通优秀人才进入非公有制企业的渠道。机关和全额事业单位的在职人员经批准自愿到非公有制企业工作的，三年内发给基本工资，鼓励吸引大中专毕业生及市内外各类人才在我市创办非公有制企业或到非公有制企业工作，在评模、评职等方面给予平等待遇。在环境上，加强和改善对非公有制企业的服务，理直气壮支持非公有制经济发展，着力营造非公有制企业留得住、引得进、能发展的良好氛围。

　　（三）深化国有企业改革，加快国有经济布局调整

　　把深化国有企业改革作为国有经济布局调整的重点，坚持百分之百触动产权、百分之百转换经营机制、百分之百转变职工身份的“三个百分之百”原则，推动国有资本从地方中小企业有序退出，力争用一到两年时间，实现地方零国有独资企业。未改制企业要一步到位，国有资本全部退出；已改制的国有控股企业，通过股权出让和吸纳其它股本，改变国有资本一股独大的局面。坚持规范运作，防止国有资产流失、债务悬空和损害职工利益等现象发生。按照国家和省统一部署，建立健全国有资产监督管理体系。完善产权交易市场，为企业产权交易创造公开公正的平台。重视已改制企业的完善提高。已改制但产权尚未彻底转移的，进一步引深改革，真正建立起现代产权制度。产权已经转移的，以股份制为主要形式，逐步加以完善。

　　（四）加大招商引资力度，全面提升对外开放水平

　　按照全市优化发展环境、促进招商引资工作的总体安排，紧贴结构调整这条主线，以发展延伸产业、新兴产业为重点、以引进项目为载体，把已掀起的招商引资热潮引向深入。今年力争达到引进资金20亿元，引进项目50个，引进高新技术不少于10项。切实加强组织领导，完善招商引资协调机制。健全招商引资机构和队伍，落实招商引资目标责任制，并将完成目标情况纳入领导干部政绩考核范畴。市政府将坚持招商引资例会制度，及时协调解决工作中遇到的矛盾和问题。下一步抓紧做好已签约项目的对接和落地工作；对前段有意向的项目及时跟踪，力求成功。坚持“走出去”与“请进来”相结合，采取专业队伍招商、经济主管部门招商、园区招商、网上招商等多种办法，进一步拓展招商引资的领域和成果。多途径宣传朔州的优势，介绍招商的政策，推荐筹备的项目，提高知名度和吸引力。市委、市政府已出台的招商引资优惠政策要坚决兑现，取信于民。

　　（五）加强基础设施建设，加快城镇化进程

　　提升城市建设与管理水平。完成城市总体规划的报批和城市近期规划、配套性专业规划的编制工作。加强规划管理，严格按规划建设城市。继续实施经营城市战略，加快市政公用行业市场化进程，盘活城市土地等资源。集中搞好水、电、路、气、热、通讯和环保七大系统建设，继续推进污水处理厂、垃圾处理厂、安泰街开发、马邑北路建设、城市新区、旧城保护与改造等一批重点工程。今年新增城市绿化面积45万平方米，使城市绿化覆盖率提高1．5个百分点，达到30％；人均公共绿地面积增加1平方米，达到16平方米。建立健全城市管理制度，落实长效管理机制，下决心解决市区的脏、乱、差问题。加强城市社区建设，强化住宅小区管理，进一步规范运作，加快步伐，扩大大物业管理覆盖面。

　　加快小城镇建设步伐。完成全市县域城镇体系规划及总体规划的编制工作。加快县城基础设施的配套完善和旧城改造，全面启动其他建制镇建设。制定优惠政策，吸引多方投入，促进小城镇产业集聚和人口集聚。

　　大力发展交通事业。加快实施“651134”工程，即建成6条重点公路（平朔一级公路、大运高速公路山阴县城连接线及怀仁毛皂出口连接线、右玉通市油路胡村至东平太段、广武至阳方口、大运路市区段改线），改造5条县际公路（109线右玉段改造、109线平鲁段改造、怀仁陈家堡至浑源界改建、平朔线改建、虎山线改造），完成11条通乡油路和300个村通油路（水泥路），建设4个汽车站场（应县、右玉、市客运北站三个汽车站及阳运物流中心）。

　　（六）强化财税金融工作，加强和改善政府宏观调控

　　贯彻落实好宏观调控的政策和措施。坚持从实际出发。区别情况，分类指导，适度调控。既要控制固定资产投资的过快增长和低水平的重复建设，又要增加固定资产投入、保持一定数量的投资规模，把全社会投资引导到国家鼓励发展、我市适宜发展的产业上。加强经济运行的协调、监测、监控，通过运用经济、法律手段和必要的行政手段，增加有效供给，保持物价总水平的基本稳定，及时消除我市经济运行中的不稳定因素。

　　继续推进财税改革和增收节支工作。深化部门预算、国库集中支付、政府采购和收支两条线管理改革。坚持依法治税，强化征管，应收尽收。严格执行支出预算，依法确保重点支出，继续执行一般性支出零增长政策，严格控制财政供养人员增长，制止各种铺张浪费行为，确保财政收支基本平衡。

　　切实改进金融服务。在当前信贷适度从紧的情况下，要积极寻求有效途径，切实改进对中小企业的信贷评估、审批和贷款制度，努力增加信贷规模，改善金融服务。支持和加强金融监管，防范和化解金融风险。稳步推进农村信用社改革。

　　整顿和规范市场经济秩序。坚持标本兼治，完善市场监管体系，形成长效监督机制，维护正常的市场秩序。重点抓好直接关系人民群众安危健康的食品、药品等专项整治，严厉打击制假售假、商业欺诈等不法行为。加快推进信用朔州建设，努力增强全社会的信用意识，不断完善激励守信、惩戒失信的社会信用监督约束机制。年内建设和完善市县两级企业信用信息网络平台。

　　（七）大力实施科教兴市战略，发展社会各项事业

　　加快推进科技进步。围绕科技兴农、科技兴企两大任务，加大科技投入和科技项目实施力度，加速推进以各类园区为载体的科技示范基地建设。加强科技培训工作，提高劳动者的科技素质。广泛开展科技交流与合作，争取更多的科技项目落户我市。新设立的市科学技术奖，每年奖励一批科技示范单位和科技带头人。

　　大力发展教育事业。兴朔州之本，唯在教育。坚持把教育工作把教育放在优先发展的地位，对各级各类教育特别是农村教育，、投入上给予更多的倾斜，工作上给予更多的支持。要普及和提升九年义务教育水平。全面启动“农村教育振兴工程”，切实做到“六个确保”，即确保义务教育经费和投入落实到位，确保教师编制定岗落实到位，确保教职工工资按时足额发放，确保校舍改造建设任务如期完成，确保农村教育质量稳步提高，确保全市农村孩子都能上学。全理调整农村学校布局，有重点地发展一批农村寄宿制小学，建设一批高标准的农村初中。右玉县要在年内通过省政府“普九”验收，怀仁县达到“义务教育标准化建设县”的要求。确保质量，扩大规模，加快发展高中教育。积极发展职业教育、成人教育。做好朔州师范升格为专科学校的有关工作，深化教育改革，全面推行中小学校长聘任制和教师全员聘用制。支持和规范民办教育和社会力量办学。大力实施人才强市战略，紧紧抓住培训、吸引、用好三个环节，不断完善选人用人、利益激励、人才流动三大机制，加强党政人才、企业经营管理人才和专业技术人才三支队伍建设，为全市各项事业发展提供有效的智力支持。

　　积极发展卫生、文化等社会事业。高度重视并加强公共卫生建设，切实抓好疾病预防控制体系、突发公共卫生事件医疗救治体系、重大疫情信息网络体系和卫生执法监督体系建设。今年市疾控中心及应县、山阴、怀仁、右玉四个县的疾控中心要全面投入使用。改革乡镇卫生院管理体制，逐步推进农村合作医疗制度。积极组织开展群众性文化活动，繁荣文化事业。不断建设完善市级文化设施和基层文化阵地，加快广播电视网络的改造与整合。完成广武旧城及汉墓群文物保护规划。积极推进全民健身运动，加快竞技体育发展步伐。强化人口与计划生育管理和服务，稳定低生育水平，努力完成年度人口控制目标。加强环境保护，大力整治违法排污企业，积极推进清洁生产，严格控制污染，城市环境空气质量要基本消灭四级和五级，努力实现整体环境质量明显好转。发展档案、史志、气象、测绘等事业。做好统计工作，搞好经济普查。关心支持老龄、妇女、儿童、残疾人、民族宗教等工作。深入开展国防教育，加强民兵预备役部队建设，做好退伍转业军人安置工作。加强社会主义精神文明建设，认真贯彻公民道德建设实施纲要，深入开展文明单位、文明村镇、文明社区等群众性创建活动。

　　各位代表，创建平安朔州、维护社会稳定，是保障全市经济社会健康和人民群众安居乐业的基本条件，是政府工作的一项重要任务。引深严打整治斗争，依法打击各种犯罪活动，深入开展同“法轮功”等邪教组织的斗争。落实社会治安综合治理的各项措施，着力改善社会治安环境。高度重视、分级负责，认真做好信访工作，及时化解矛盾，协调利益关系，努力把问题解决在基层和萌芽状况。把安全生产摆到更加突出的位置，做到警钟长鸣，常抓不懈，超前防范，严格执法，全力控制各类事故特别是重特大事故的发生。

　　（八）完善社会保障体系，努力改善群众生活

　　加强社会保障，老生常谈肋弱势群体，是人民之盼，政府之责，社会之德。要时刻关心人民群众特别是弱势群体的生活，多为他们献爱心，解忧愁，送去党和政府的温暖。

　　千方百计增加就业。把增加就业放到政府工作的突出位置，全面落实中央、省、市促进就业再就业的各项政策措施，大力开发就业岗位，重视发展劳动密集型产业、中小企业、第三产业特别是社区服务业，力争全年净增就业岗位1．3万个。继续实施再就业援助制度，政府出资开发公益性岗位，优先安置“4050”等就业困难人员。加强街道社区劳动保障平台建设，完善就业服务体系，做好就业指导和培训工作，促进下岗失业人员多形式就业。

　　加快完善社会保障体系。确保下岗职工基本生活费和离退休人员养老金按时足额发放。健全失业保险制度，妥善解决下岗职工出“中心”遇到的问题。完善城镇职工基本医疗保险制度，启动职工生育保险和工伤保险。城市居民最低生活保障制度要切实做到应保尽保。扩大各类社会保险的覆盖面，重点拓展非公有制企业。加强社会保险费的收缴、管理工作。积极开展社会救助活动。

　　增加中低收入群体的收入。多渠道增加农民现金收入，加大对拖欠农民工工资问题的检查和处罚力度，力争在年内解决农民工工资历史拖欠80％以上，并不再发生新的拖欠。各类企业都要按时支付工资，严格执行最低工资制度。认真落实中央和省的增资政策，确保公教人员工资按时足额发放。

　　各位代表，坚持执政为民，努力维护好、发展好、实现好广大人民群众的根本利益，是政府一切工作的根本出发点和最终归属。今年，市人民政府要为人民群众办好八项“民心工程”：一是解决农村8万人的饮水困难。二是完成村村通油路（水泥路）600公里，力争全市基本实现村村通油路（水泥路）。三是搞好农村沼气开发利用。全市新建沼气综合利用模式大棚5000座，其中怀仁、应县、右玉每个县建设1500座以上。四是全面启动农村低保，解决农村低保对象2780人的基本生活问题。五是搞好怀仁县农村新型合作医疗试点。六是改造138所农村危房学校。七是在市区新建一到两所中小学校。八是建设市中心医院，年内一期工程竣工。以上八项“民心工程”，是年初市政府向全市人民作出的庄严承诺，现都已启动，一定要说到做到，办实办好！

　　三、加强政府自身建设，努力创优发展环境

　　我们各级政府必须牢固树立一切为经济社会发展服务、一切为优化环境服务的思想，努力学习，转变观念，转变职能，转变作风，进一步强化自身建设，改善和提升政府形象，加快形成“政府创造环境、企业创造文明、人民创造财富”的良好局面。

　　第一，求真务实，改进作风，建设勤政为民的政府。各级政府领导要强化宗旨意识，想群众之所想，急群众之所急，办群众之所需，尽心竭力办好事关群众切身利益的事情，尤其是要以更多的感情投入，更多的关爱行动，去帮助解决农村贫困人口、城市下岗失业人员等弱势群体的生产生活困难。大力弘扬求真实务的精神，讲实话，办实事，求实效，深入基层，深入群众，经常到条件差、困难多的地方去，体察民情，关注民生，解决问题。不断创新完善领导工作目标责任制，每项工作都要做到目标明确，责任明确，做到督促、考核、奖惩到位。牢固树立正确的政绩观，坚决反对官僚主义和形式主义，坚决反对做表面文章，决不搞劳民伤财的“形象工程”和“政统工程”，真正把人民的满意程度作为政府工作根本的评定标准。

　　第二，转变职能，优化服务，建设施政有为的政府。深化行政管理体制税管理方式改革，建设服务型政府。加速政企、政事、政资分开，退出越位，纠正错位，补足缺位，真正将政府职能转到经济调节、市场监管、社会管理和公共服务上来，转到创造良好的发展环境上来。加快建立健全各种突发事件应急机制，提高政府应对公共危机的能力。实施政府“提速”工程，继续推进电子政务，提高行政效率。市政务大厅和县级政务中心要完善运行机教，提升服务水平。实施“阳光”行政，建立政务信息公开制度。在全市开展“争当人民满意公务员”活动，强化公务员的服务意识，努力创造“支持改革、鼓励干事、保护创业、注重服务”的软环境，使朔州大地成为投资的乐土、发展的热土、创业的乐园。

　　第三，科学决策，依法行政，建设民主法制的政府。完善公众参与、专家论证和政府决策相结合的决策机制，加快建立和完善重大问题集体决策制度、专家咨询制度、社会公示和社会听证制度、决策责任追究制度。强化决策执行监督，确保政令畅通、决策落实。各级政府要按照法定权限和程序行使权力，履行职责，规范行为。全面贯彻实施《行政许可法》，认真清理行政许可的项目、规定和实施主体，并将清理结果公布于众。强化行政执法监督，实行行政执法责任制和执法过错追究制，做好行政复议、行政应诉、行政赔偿工作，努力做到有权必有责、用权受监督、侵权要赔偿。各级政府要自觉接受同级人大及其常委会的监督，认真执行人大的决议，定期报告政府工作。积极接受人民政协的民主监督，认真听取民主党派、工商联、无党派人士和各人民团体的意见。拓宽新闻舆论、社会公众监督等渠道，确保人民赋予的权力真正服务于人民。

　　第四，从严治政，严格自律，建设清正廉洁的政府。我们要始终牢记“两个务必”，坚决贯彻执行有关廉洁自律的各项规定，全面落实领导干部党风廉政建设责任制，做到守本份，讲廉洁，反奢侈，讲奉献。加大治本力度，严格执行领导干部离任审计和任期经济责任审计制度，健全国有土地出让、建设项目招投标、财政收支等制度，从源头上、机制上预防腐败。继续做好“三项治理”工作。坚决查处违法违纪案件，不断惩治政府公务员中的腐败分子。坚持纠、评、建结合，加强行风建设，坚决纠正损害人民群众利益的行业歪风。严格行政工作纪律，依法改善行政执法环境，重点整治乱收费、乱罚款、乱摊派、乱检查的“四乱”行为，坚决惩处行政“不作为”和“乱作为”的人与事。我们要以党风廉政建设的新成果真正取信于民！

　　各位代表，新一届政府肩负着全面建设小康社会的历史重任，寄托着全市人民的殷切希望。我们面临的任务艰巨而光荣，我们的明天灿烂而辉煌。让我们高举邓小平理论伟大旗帜，认真实践三个代表重要思想，在中共朔州市委的坚强领导下，紧紧依靠全市人民的智慧和力量，抢抓新机遇，迎接新挑战，拓展新空间，创造新业绩，在全面建设小康社会的伟大实践中取得新的更大的成就！
